# The Cervical Dystonia Impact Profile (CDIP-58): Can a Rasch developed patient reported outcome measure satisfy traditional psychometric criteria?

**DOI:** 10.1186/1477-7525-6-58

**Published:** 2008-08-06

**Authors:** Stefan J Cano, Thomas T Warner, Alan J Thompson, Kailash P Bhatia, Ray Fitzpatrick, Jeremy C Hobart

**Affiliations:** 1 Neurological Outcome Measures Unit, Institute of Neurology, University College London, Queen Square, London, UK; 2 Department of Clinical Neuroscience, Room N16 ITTC Building, Peninsula College of Medicine and Dentistry, Tamar Science Park, Davy Road, Plymouth, UK; 3 Department of Clinical Neurosciences Royal Free & University College Medical School, London, UK; 4 Sobell Department of Motor Neuroscience and Movement Disorders, Institute of Neurology, University College London, Queen Square, London, UK; 5 Department of Public Health, University of Oxford, Old Road Campus, Roosevelt Drive, Headington, Oxford, UK

## Abstract

**Background:**

The United States Food and Drug Administration (FDA) are currently producing guidelines for the scientific adequacy of patient reported outcome measures (PROMs) in clinical trials, which will have implications for the selection of scales used in future clinical trials. In this study, we examine how the Cervical Dystonia Impact Profile (CDIP-58), a rigorous Rasch measurement developed neurologic PROM, stands up to traditional psychometric criteria for three reasons: 1) provide traditional psychometric evidence for the CDIP-58 in line with proposed FDA guidelines; 2) enable researchers and clinicians to compare it with existing dystonia PROMs; and 3) help researchers and clinicians bridge the knowledge gap between old and new methods of reliability and validity testing.

**Methods:**

We evaluated traditional psychometric properties of data quality, scaling assumptions, targeting, reliability and validity in a group of 391 people with CD. The main outcome measures used were the CDIP-58, Medical Outcome Study Short Form-36, the 28-item General Health Questionnaire, and Hospital and Anxiety and Depression Scale.

**Results:**

A total of 391 people returned completed questionnaires (corrected response rate 87%). Analyses showed: 1) data quality was high (low missing data ≤ 4%, subscale scores could be computed for > 96% of the sample); 2) item groupings passed tests for scaling assumptions; 3) good targeting (except for the Sleep subscale, ceiling effect = 27%); 4) good reliability (Cronbach's alpha ≥ 0.92, test-retest intraclass correlations ≥ 0.83); and 5) validity was supported.

**Conclusion:**

This study has shown that new psychometric methods can produce a PROM that stands up to traditional criteria and supports the clinical advantages of Rasch analysis.

## Background

Patient reported outcome measures (PROMs) are increasingly being used as primary or secondary outcome measures in clinical trials [1,2]. As such, the quality of inferences made from clinical trials is dependent on the PROMs used. This increasingly acknowledged fact has led the United States Food and Drug Administration (FDA) to produce guidelines [3,4] that specify minimum criteria for the scientific adequacy of scales in clinical trials. These are likely to be followed by the European Medicines Agency (EMEA) [5], and will have implications for all scales used in future clinical trials.

The Cervical Dystonia Impact Profile (CDIP-58) assesses the health impact of CD [6]. It was developed using new, sophisticated, but not widely known techniques of PROMs construction (Rasch analysis), which are, as of yet, not included in the FDA guidelines. In addition, researchers interested in using the CDIP-58, who may be unfamiliar with new psychometric methods, may find the original paper [6] abstruse and intangible.

The aim of this study is to provide clinicians with a comprehensive evaluation of the CDIP-58 using a traditional approach to scale evaluation for three reasons: 1) provide traditional psychometric evidence for the CDIP-58 in line with the proposed FDA guidelines; 2) enable researchers and clinicians to make their own judgment of its performance and compare it with existing dystonia scales; and 3) help researchers and clinicians bridge the knowledge gap between old and new reliability and validity testing methods.

## Methods

### Setting and Participants

A random sample of 460 people with CD was recruited from a complete list of 1110 members from the Dystonia Society of Great Britain. The sampling strategy is described elsewhere [6]. A booklet of questionnaires was administered as a postal survey following standard techniques [7]. In addition, 140 individuals were randomly selected to receive a second identical battery after 2 weeks to estimate test-retest reproducibility (TRT). This study was reviewed and passed by the local hospital trust research ethics committee.

### Measurement model

**Figure 1:**
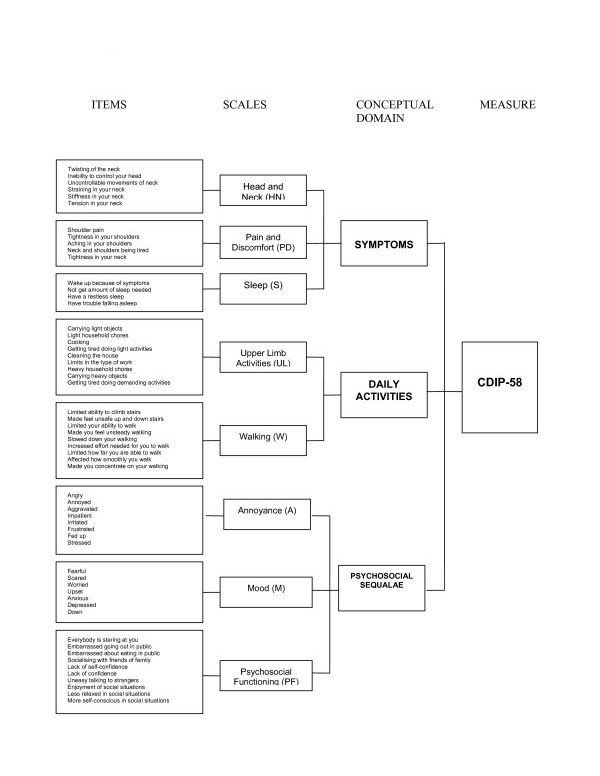
Measurement model of the CDIP-58.

In the traditional psychometric paradigm, a measurement model proposes how items in a measure are grouped into scales, and in turn how scales are scored. This definition of a measurement model is different to that in the Rasch measurement paradigm, which instead views it as a formulation that represents the structure which data should exhibit in order to obtain measurements from the data. The CDIP-58 measurement model groups the 58 items into eight subscales: head and neck symptoms (6 items), pain and discomfort (5 items), upper limb activities (9 items), walking (9 items), sleep (4 items), annoyance (8 items), mood (7 items), and psychosocial functioning (10 items) [6]. We examined whether the model (Figure 1) fulfilled fundamental prerequisites for rigorous measurement as defined by traditional psychometric approaches [8,9].

### Data analyses

CDIP-58 subscale item responses were summed without weighting or standardisation to generate scores [10]. Each subscale score was transformed to have a common range of 0 (no impact) to 100 (most impact) [11]. Five psychometric properties were examined: data quality, scaling assumptions, targeting, reliability and validity. Table 1 shows the extent to which the CDIP-58 testing conforms to the draft guidelines proposed by the FDA [3,4].

**Table 1:** Adapted from table 4 of the FDA draft guidelines for measurement properties reviewed for PRO instruments used in clinical trials

**Measurement Property**	**Test**	**Methods used in testing the CDIP-58**
Reliability	Test-retest	✓
Internal consistency	Whether the items in a domain are intercorrelated, as evidenced by an internal consistency statistic (e.g., coefficient alpha)	✓
Inter-interviewer reproducibility (for interviewer-administered PROs only)	Agreement between responses when the PRO is administered by two or more different interviewers	NA
Validity	Content-related	✓*
Ability to measure the concept (also known as construct-related validity; can include tests for discriminant, convergent, and known-groups validity)	Whether relationships among items, domains, and concepts conform to what is predicted by the conceptual framework for the PRO instrument itself and its validation hypotheses.	✓
Ability to predict future outcomes (also known as predictive validity)	Whether future events or status can be predicted by changes in the PRO scores	✗
Ability to detect change	Includes calculations of effect size and standard error of measurement among others	✓**
Interpretability	Smallest difference that is considered clinically important; this can be a specified difference (the minimum important difference (MID)) or, in some cases, any detectable difference. The MID is used as a benchmark to interpret mean score differences between treatment arms in a clinical trial	✓/✗***
Responder definition – used to identify responders in clinical trials for analyzing differences in the proportion of responders between treatment arms	Change in score that would be clear evidence that an individual patient experienced a treatment benefit. Can be based on experience with the measure using a distribution-based approach, a clinical or non-clinical anchor, an empirical rule, or a combination of approaches.	NA

✓ = tested; ✗ = not tested; * Reported in Cano et al 2004 [6]; ** Reported in Cano et al 2006 [28]; *** Although not including MID in our responsiveness paper (Cano et al, 2006 [28]), we include a comparison of relative responsiveness to existing PROs used in CD research in order to increase the interpretability of CDIP-58 change scores against these measures.

#### Data quality

Data quality concerns the completeness of item- and scale-level data, and was determined by the percentage of missing data for items, and the percentage of computable scale scores [8]. The criterion for acceptable item-level missing data was < 10% [12] and for computable scale scores > 50% completed items [13].

#### Scaling assumptions

Three scaling assumptions should be satisfied for scale scores to be generated using the proposed item groups, and Likert's method of summated ratings [14,15].

1. Items in each scale should measure a common underlying construct otherwise it is not appropriate to combine them to generate a scale score [16]. This was evaluated by examining the correlation between each item and scale score computed from the remaining items in that scale (corrected item-total correlation). The criterion used was corrected item-total correlation ≥ 0.30 [17].

2. Items in each scale should contain a similar proportion of information concerning the construct being measured otherwise they should be given different weights [10]. This criterion was determined by examining the equivalence of corrected item-total correlations. The criterion used was corrected item-total correlation ≥ 0.30 [17].

3. Items should be correctly grouped into scales. That is, items should correlate higher with the total score of their own scales (item own-scale correlation) than with the total score of the other scales (item other-scale correlations). The recommended criterion for definite scaling successes are item-own scale correlations (corrected for overlap) exceeding item-other scale correlations by at least two standard errors (2 × 1√n) [17]. In situations where this criterion was not reached, we examined the magnitude of differences between item-own and item-other scale correlations. The greater the magnitude of differences between item-own scale and item other-scale correlations, the greater the support for scaling success.

#### Targeting

The targeting of a scale to a sample indicates whether a scale is acceptable as a measure for the sample. It is recommended that: scale scores should span the entire scale range; floor (proportion of the sample at the maximum scale score) and ceiling (proportion of the sample at the minimum scale score) effects should be low (<15%) [18]; and skewness statistics ranging should range from -1 to +1 [19].

#### Reliability

Reliability is the extent to which scale scores are dependable and consistent. Two types were examined. Internal consistency, reported as Cronbach's alpha coefficients, estimates the random error associated with scores from the intercorrelations among the items [20]. TRT reproducibility, reported as intraclass correlations coefficients (ICC) on scores produced by a sub sample assessed twice over a 2-week interval, estimates the ability of CDIP-58 subscales to produce stable scores over a given period of time in which the respondents' condition is assumed to have remained the same [19]. We used a two-way random effects model based on absolute agreement as a suitable, conservative estimate of test retest reliability, as this type of ICC accounts for the systematic differences among levels of ratings. This is because the raters used were only a sample of all possible raters. We used a two-way random effects model based on absolute agreement as a suitable conservative estimate test retest reliability, as this type of ICC accounts for the systematic differences among levels of ratings [21]. Recommended criteria for adequate reliability are Cronbach's alpha coefficient ≥ 0.80 [21], and TRT ICC ≥ 0.80 [22].

#### Validity

Validity is the extent to which a scale measures what it intends to measure and is essential for the accurate and meaningful interpretation of scores [23]. Three aspects were tested:

1. Intercorrelations between CDIP-58 subscales were assessed to examine the extent to which scales measured separate but related constructs [8]. The magnitude of intercorrelations between CDIP-58 subscale scores were predicted to be consistent with expectation about the proximity of the constructs, and were generally expected to be moderate in size (r = 0.30–0.70) [24]. In addition, subscale reliabilities should be larger that inter-scale correlations to support that scales measure distinct constructs.

2. Correlations between CDIP-58 subscales and other scales were examined. Patients were asked to complete three other questionnaires for validity testing: Medical Outcome Study 36-item Short Form Health Survey (SF-36) measures health status in eight multi-item scales (Role Limitations-Emotional, Role Limitations-Physical, Bodily Pain, Vitality, General Health Perceptions, Social Functioning, Physical Functioning, Mental Health) [25]; 28-item version of the General Health Questionnaire (GHQ-28) measures psychological well being in four dimensions (Somatic Symptoms, Anxiety, Social, Depression) [26], and Hospital and Anxiety and Depression Scale (HADS) measures mood in two scales (Depression and Anxiety) [27]. A number of hypotheses were made based on the direction, magnitude and pattern of correlations being consistent with expectations based on the proximity of the constructs.

Ideally for the results of correlations between CDIP-58 subscales and other scales to be fully interpretable the external measures should be reliable and valid. Whereas we have previously examined the psychometric properties of the SF-36 in CD [9], there are no current published articles which have examined the HADS or GHQ-28. Our reasoning for selecting the latter two scales was on the basis of their wide-spread use in neurologic research. Importantly, this is a common limitation of reliability and validity testing and the findings should be interpreted with this borne in mind.

Criteria were used as guides as to the magnitude of correlations, as opposed to pass/fail benchmarks (high correlation r > 0.70 and moderate correlation r = 0.30–0.70):

a. The Pain and Discomfort subscale would correlate more highly with the SF-36 bodily pain than with unrelated measures of psychological functioning (SF-36 Mental Health, HADS Anxiety and Depression).

b. The Upper Limb and Walking subscales would correlate more highly with the SF-36 physical functioning than with unrelated measures of psychological functioning (SF-36 Mental Health, HADS Anxiety and Depression, GHQ-28).

c. The Annoyance and Mood subscales would correlate more highly with the SF-36 Mental Health than with unrelated measures of physical functioning (e.g. SF-36 physical functioning).

d. The Annoyance and Mood subscales would correlate moderately with the HADS, GHQ-28 anxiety and depression scales as these reflect aspects of mood.

e. The Psychosocial Functioning subscale would correlate moderately with the SF-36 social functioning as this reflects an aspect of psychosocial functioning.

3. Correlations between CDIP-58 subscales and sociodemographic variables (age, sex, and level of education attained) were examined to determine the extent to which they were biased by these variables. We predicted that these correlations would be low < 0.30.

## Results

### Sample

Of the 460 patients who received the CDIP-58, 391 returned completed questionnaires (corrected response rate = 87%). Of the 140 TRT questionnaires, 105 were returned completed (corrected response rate = 75%). The sample included people with a wide range of ages and disease duration (Table 2) [6].

**Table 2:** Respondent characteristics

Variable*	*Sample*
Number	391
Sex	
Female	72
Age	
Mean (SD)	*58 (12)*
Range	*25–88*
Ethnicity	
White	*97*
Years since CD onset	
Mean (SD)	*15 (10)*
Range	*2 – 50*
Employment status	
Retired	35
Employed	29
Unable to work due to CD	20
Treatment	
Botulinum Injections	90
Drug therapy	53
Alternative treatment	0
Surgery	0
External measures (Mean; SD)	
SF-36** Bodily Pain	46 (26)
SF-36 Social Functioning	55 (31)
SF-36 Physical Functioning	57 (29)
SF-36 Mental Health	62 (20)
	
GHQ*** Anxiety	38 (19)
GHQ Depression	14 (19)
	
HADS*** Anxiety	43 (24)
HADS Depression	33 (19)

*All values are percentages unless specified otherwise; ** range 0-100,  *** converted to 0-100 (original range 0-21)

### Psychometric properties

#### Data quality (Table 3)

**Table 3:** Data quality, scaling assumptions, targeting, reliability and validity

**CDIP Scale**	Head and Neck Symptoms (6 items)	Pain and Discomfort (5 items)	Upper Limb Activities (9 items)	Walking (9 items)	Sleep (4 items)	Annoyance (8 items)	Mood (7 items)	Psycho- social Functioning (10 items)
**Psychometric property**								
Data quality								
Item missing data (range %)	1–2	3–4	2–4	3–4	2	2–4	2–4	1–2
Computable scale scores (%)	98	97	98	97	98	98	96	99
								
Corrected item-total correlations								
Mean	0.76	0.82	0.78	0.87	0.89	0.83	0.78	0.82
Range	0.67–0.81	0.70–0.87	0.64–0.87	0.82–0.91	0.84–0.93	0.79–0.89	0.68–0.84	0.70–0.90
Item-other scale correlations								
Range	0.37–0.72	0.40–0.75	0.43–0.72	0.42–0.75	0.39–0.52	0.42–0.73	0.39–0.82	0.38–0.69
Scaling successes (%)	83	80	100	100	100	100	86	100
								
Targeting								
Mean score	57.6	53.8	42.9	37.1	33.3	37.9	29.7	49.2
Standard deviation	25.6	27.9	27.6	31.5	31.5	26.9	24.9	29.5
Score range	0–100	0–100	0–100	0–100	0–100	0–100	0–100	0–100
Floor/ceiling effect (%)	1/6	2/7	7/0	17/4	27/7	7/4	13/1	5/4
Skewness	-0.23	-0.15	0.11	0.44	0.66	0.51	0.82	0.02
								
Reliability (n = 377–385)								
Cronbach's alpha	0.92	0.93	0.94	0.97	0.96	0.96	0.95	0.96
TRT (ICC; n = 92–95)	0.85	0.83	0.94	0.95	0.86	0.83	0.85	0.89

Data quality was high. The proportion of item-level missing data was low (≤ 4%). Subscale scores could be computed for at least 96% of the sample.

#### Scaling assumptions (Table 3)

Item groupings in each of the eight CDIP-58 subscales passed tests for scaling assumptions:

1. Corrected item-total correlations for each of the eight CDIP subscales ranged from 0.64–0.93 satisfying the recommended criteria (> 0.30). This supported that items in each subscale of the CDIP-58 measured a common underlying construct.

2. Corrected item-total correlations > 0.30 indicated that items in each of the subscales contained a similar proportion of information.

3. Fifty-five of the fifty-eight items correlated higher with their own subscale than other subscales. Forty-seven of these exceeded the criterion (2 × 1√n). This provided some support for the grouping of items in each of the eight subscales. There was less support for three items which correlated higher with other subscales: Head Neck symptoms 'stiffness in your neck' (Pain and Discomfort subscale, r = 0.72), Pain and Discomfort 'tightness in your neck' (Head and Neck symptoms subscale, r = 0.75), and Upper Limb 'getting tired when doing demanding physical activities' (Walking subscale, r = 0.82).

#### Targeting (Table 3)

Subscale scores spanned the entire scale range. However, the Walking scale fell just outside of the criterion (ceiling effect = 17%) and the Sleep subscale was found to have a more significant ceiling effect (27%). Despite this, responses were not notably skewed (-0.23 to +0.82). These findings indicate good scale-to-sample targeting, thus supporting total and subscale scores as appropriate for all patients representing the full spectrum of CD impact.

#### Reliability (Table 3)

Cronbach's alpha, and test-retest ICCs for all eight CDIP-58 subscales were high (> 0.83), supporting their reliability.

#### Validity (Table 4)

**Table 4:** Convergent and discriminant construct validity of the CDIP-58

Instrument	Scale/ Dimension/ Variable								
	Validity (Correlation)	Head and Neck Symptoms (6 items)	Pain and Discomfort (5 items)	Upper Limb Activities (9 items)	Walking (9 items)	Sleep (4 items)	Annoyance (8 items)	Mood (7 items)	Psycho- social Funct-ioning (10 items)

CDIP-58	Head and Neck Symptoms								
	Pain and Discomfort	**0.72^a^**	-						
	Upper Limb Activities	0.64	0.53	-					
	Walking	0.63	0.65	**0.79^a^**	-				
	Sleep	0.50	0.53	0.54	0.50	-			
	Annoyance	0.63	0.55	0.60	0.56	0.52	-	-	
	Mood	0.52	0.48	0.54	0.53	0.49	**0.84^a^**	-	
	Psychosocial Functioning	0.67	0.51	0.55	0.53	0.44	**0.73^a^**	0.69	-
									
SF-36*	Bodily Pain	-0.60	**-0.71^b^**	-0.72	-0.62	-0.59	-0.54	-0.51	-0.48
	Social Functioning	-0.56	-0.56	-0.70	-0.62	-0.55	-0.63	-0.59	**-0.69^b^**
	Physical Functioning	-0.45	-0.57	**-0.80^b^**	**-0.78^b^**	-0.53	**-0.43^b^**	**-0.43^b^**	-0.45
	Mental Health	-0.43	**-0.41^b^**	**-0.72^b^**	**-0.40^b^**	-0.43	**-0.73^b^**	**-0.78^b^**	-0.60
									
HADS	Anxiety	0.34	**0.39^b^**	**0.31^b^**	**0.28^b^**	0.31	**0.54^b^**	**0.66^b^**	0.48
	Depression	0.41	**0.45^b^**	**0.52^b^**	**0.51^b^**	0.42	**0.59^b^**	**0.60^b^**	0.50
									
GHQ**	Anxiety	0.30	**0.30^b^**	**0.34^b^**	**0.19^b^**	0.22	**0.44^b^**	**0.50^b^**	0.30
	Depression	0.35	**0.29^b^**	**0.31^b^**	**0.36^b^**	0.18	**0.53^b^**	**0.66^b^**	0.43
									
Demo-graphic	Age	-0.05	-0.03	-0.02	0.01	0.00	-0.04	-0.07	0.00
Variables	Sex	-0.04	-0.17	0.02	-0.07	0.00	0.06	0.04	0.02
	Education	-0.04	-0.05	-0.01	-0.07	-0.02	-0.03	-0.03	-0.08

*4/8 scales omitted from table as not applicable to analyses; **2/8 scales omitted from table as not applicable to analyses

^a^Correlations falling outside of the predicted range; ^b^Correlations consistent with predictions

1. Intercorrelations between CDIP-58 subscales ranged from 0.44 – 0.84, suggesting the subscales measured related but different constructs. A few of the correlations fell outside of the predicted range of correlations, and were highly correlated (highlighted in Table 2). However, the correlations were not unreasonable given the proximity of the constructs in each of the subscales (see Figure 1) and scale reliabilities were larger than inter-scale correlations supporting that CDIP-58 subscales measure distinct constructs.

2. Correlations between CDIP-58 subscales and hypothesised related scales of the SF-36, GHQ and HADS were consistent with predictions (highlighted in Table 4). This provides support that the CDIP-58 subscales measure what they intend to measure.

3. Correlations between CDIP-58 subscales and sociodemographic variables (age, sex, and level of education attained) were in general low (-0.17 to +0.06). This finding suggests that responses to the CDIP-58 were not biased by socio-demographic factors.

## Discussion

The forthcoming FDA guidelines make it increasingly important for researchers and clinicians to be exposed to the science behind PROMs. In this study, the CDIP-58 satisfied traditional psychometric criteria for data quality, scaling assumptions, targeting, reliability and validity. We hope that this, together with our previous work on conceptual model and scale development [6] and assessment of the sensitivity to clinical change of the CDIP-58 following Botulinum Toxin Type A (that found it to be superior to existing CD PROMs [28]), provides an evidence-base for its use in clinical trials, in line with the forthcoming FDA guidelines. As such, the CDIP-58 offers an advance on current PROMs. In addition, our findings are relevant to practicing neurologists, who can use this information to compare the CDIP-58 to existing published CD PROM data, which will help to avoid an ad hoc approach which may negatively impact upon rigorous measurement.

Three main issues arise from the findings. First, were there any instances where traditional psychometric criteria were not met and how should we interpret these? Second, how can the information provided here be used and what do traditional psychomteric analyses tell us? Third, what is the added value of using Rasch analysis to develop PROMs and in particular, what benefits are gained from the required additional investment in skill level, retraining and software costs?

Traditional psychometric analyses detected one problem not identified by Rasch analysis. Tests of scaling assumptions were failed by 3 items ('stiffness in the neck', 'tightness in the neck', 'upset'). This means that they correlated similarly with their own subscales and other subscales they were not intended to belong in. There are three reasons why this may be the case. First, the subscales in question were themselves highly correlated. Second, these items may be non-specific indicators of their intended construct. Third, any item can exist conceptually in more than one scale. The clinical implications of this are probably minimal, as the constructs measured by the three subscales in question are anchored by the other items, which in turn performed well psychometrically.

So, how can the information provided here be used and what do traditional psychomteric analyses tell us? Researchers who are unfamiliar with Rasch analysis can use the information presented here to compare the CDIP-58 to existing published CD PROM data. The caveat is that any inferences made from this paper alone are constrained by the sample and scale limitations inherent to all studies that use traditional psychometric analyses. These include three main points. First, total scores are often analysed as if they were interval measures. However, it has been widely demonstrated that they are not, and therefore, they are not measuring consistently across the range of the scale. Importantly, we do not know the extent to which they are measuring inconsistently across the scale. Second, traditional psychometric analyses rely directly on the items and samples used to estimate them. This means that item properties vary depending on the sample and patient scores in turn depend on the set of items taken. Thus, the reliability and validity estimates of a measure may differ across different patient groups. Third, it is recommended that total scores are only used for group comparison studies and not individual patient measurement, because the confidence intervals around individual patient scores are so wide [18].

Our study suggests that Rasch analysis can produce a reliable and valid measure as defined by traditional criteria. What then is the added value of using new psychometric methods? First, when scales are successfully developed using Rasch analysis it is possible to transform ordinal level scale scores into interval level measurements [29-31]. This improves the accuracy with which we can measure differences between people and clinical change. Second, Rasch analysis enables estimates suitable for individual person measurement. This can help directly inform upon patient monitoring, management and treatment for patients. Third, reliability and validity estimates computed using Rasch analysis are much less sample dependent than those derived from traditional methods. In addition, Rasch measurement methods afford more sophisticated analyses to test theoretically driven concepts and therefore provide empirical evidence for properties such as construct validity. This has important relevance for the generalisability of PROM evaluations. These benefits are further explored in other relevant articles and texts [1,2,32-34].

We envisage that this article, in conjunction with our previous articles on the Rasch development of the CDIP [6] and our recent review in Lancet Neurology [2] describing traditional and new psychometric techniques, can be used by researchers and clinicians to help bridge the knowledge gap between traditional and modern reliability and validity testing methods. This study has shown that Rasch analysis methods can produce a PROM that stands up to traditional psychometric criteria. A demonstration of this nature is rare. It is much more common that scales developed using traditional methods to be tested ***post hoc ***using new approaches [35]. Nevertheless, direct comparisons of new and traditional psychometric methods of any nature in the medical literature are sparse, and at best superficial [36,37] In part, this may be due to the fact that these two approaches cannot be compared easily as they use different methods, produce different information, and apply different criteria for success and failure. Both approaches have their supporters and traditional psychometric methods remain the dominant paradigm. However, we believe that state-of-the-art clinical trials and research would benefit from the advantages offered by Rasch analysis.

## Conclusion

This study has shown that new psychometric methods can produce a PROM that stands up to traditional criteria and supports the clinical advantages of Rasch analysis. In addition, the CDIP-58 satisfied traditional reliability and validity criteria further supporting it as a clinically useful measure for use in routine practice, audit and treatment trials.

## Competing interests

The authors declare that they have no competing interests.

## Authors' contributions

SC collected, conducted, analysed and interpreted the data and wrote the manuscript. JH conceived and designed the study and contributed to the interpretation of data and writing of the manuscript. TW, RF, KB and AT were involved in guiding the study including design and acquisition of data, and reviewing drafts of this manuscript. All authors read and approved the final manuscript.
